# Refining the Global Phylogeny of Mitochondrial N1a, X, and HV2 Haplogroups Based on Rare Mitogenomes from Croatian Isolates

**DOI:** 10.3390/genes14081614

**Published:** 2023-08-12

**Authors:** Dubravka Havaš Auguštin, Jelena Šarac, Maere Reidla, Erika Tamm, Blaženka Grahovac, Miljenko Kapović, Natalija Novokmet, Pavao Rudan, Saša Missoni, Damir Marjanović, Marina Korolija

**Affiliations:** 1 Centre for Applied Bioanthropology, Institute for Anthropological Research, Ljudevita Gaja 32, 10000 Zagreb, Croatia; dhavas@inantro.hr (D.H.A.);; 2 Institute for Anthropological Research, 10000 Zagreb, Croatia; 3 Institute of Genomics, University of Tartu, 50090 Tartu, Estonia; 4 School of Medicine, University of Rijeka, 51000 Rijeka, Croatia; 5 Croatian Academy of Sciences and Arts, 10000 Zagreb, Croatia; 6 Faculty of Dental Medicine and Health, J. J. Strossmayer University, 31000 Osijek, Croatia; 7 Genetics and Bioengineering Department, International Burch University, 71000 Sarajevo, Bosnia and Herzegovina; 8 Forensic Science Centre “Ivan Vučetić”, Ministry of the Interior, 10000 Zagreb, Croatia

**Keywords:** rare mitochondrial haplotypes, mitochondrial phylogeny, mitochondrial haplogroup HV2b, N1a, X1-3

## Abstract

Mitochondrial DNA (mtDNA) has been used for decades as a predominant tool in population genetics and as a valuable addition to forensic genetic research, owing to its unique maternal inheritance pattern that enables the tracing of individuals along the maternal lineage across numerous generations. The dynamic interplay between evolutionary forces, primarily genetic drift, bottlenecks, and the founder effect, can exert significant influence on genetic profiles. Consequently, the Adriatic islands have accumulated a subset of lineages that exhibits remarkable absence or rarity within other European populations. This distinctive genetic composition underscores the islands’ potential as a significant resource in phylogenetic research, with implications reaching beyond regional boundaries to contribute to a global understanding. In the initial attempt to expand the mitochondrial forensic database of the Croatian population with haplotypes from small isolated communities, we sequenced mitogenomes of rare haplogroups from different Croatian island and mainland populations using next-generation sequencing (NGS). In the next step and based on the obtained results, we refined the global phylogeny of haplogroup N1a, HV2, and X by analyzing rare haplotypes, which are absent from the current phylogenetic tree. The trees were based on 16 novel and 52 previously published samples, revealing completely novel branches in the X and HV2 haplogroups and a new European cluster in the ancestral N1a variant, previously believed to be an exclusively African–Asian haplogroup. The research emphasizes the importance of investigating geographically isolated populations and their unique characteristics within a global context.

## 1. Introduction

Isolation, whether geographical or cultural, has always had an impact on the population genetic structure, and it is mirrored in the reduction of genetic diversity due to genetic drift and a higher rate of marriage in consanguinity due to the limited relation options [1]. These factors can have a favorable influence on the analyses of microevolutionary processes and population differentiation in isolated communities such as island populations or remote mountainous villages also known as “inland islands” [2,3,4]. Mitochondrial DNA (mtDNA) is used in such population-genetic research due to its maternal inheritance that can trace individuals along the maternal lineage across many generations in the past. Scenarios involving genetic drift, repeated bottlenecks, and the founder effect, together with marriage in consanguinity, could explain how the Adriatic islands’ mtDNA pool accumulated a subset of lineages that are almost absent in other European populations, clearly indicating that the islands can be very useful in genetic variability research, even on a global scale [5,6,7,8]. Indeed, other genetic studies on isolated European populations revealed certain private mtDNA motifs, explaining their genetic ancestry and the historical events that have shaped their genetic structure [9,10]. 

Mitochondrial DNA is also extensively used in forensic casework to characterize biological evidence based on its specific features, such as high copy numbers within cells and the small size of the mitochondrial genome, also very useful in analysis of ancient human remains. The currently available forensic mtDNA database is the EMPOP database, specifically developed for forensic applications, and comprising around 48,500 quality controlled mitotypes (URL: https://empop.online/, accessed on 28 June 2023). However, the number of complete mitogenomes in EMPOP (around 4000) is still insufficient and lacks contributions from Croatia [11,12]. In the initial attempt to expand the current Croatian forensic database and achieve a fully representative database of the Croatian territory, we randomly selected samples belonging to rare mtDNA haplogroups (<5% in the general European population), mostly from island and mountainous populations, to be sequenced on the Illumina platform. To the best of our knowledge, several sequenced mitogenomes presented a novelty not only in the current Croatian database but also in the global mtDNA literature. Those samples belonged to a rare variant of haplogroup N1a, harboring the ancestral 16147G mutation, samples of unknown subhaplogroup affiliation in the X phylogeny, and samples belonging to the HV2 haplogroup that share the defining mutations for the HV2a haplogroup but otherwise belong to a completely different clade that we named HV2b.

The mitochondrial haplogroups N1a and X originated most likely in the Arabian Peninsula and the Near East. Although very rare and with uneven distributions in contemporary populations, they harbor a very large diversity of lineages across western Eurasia and Africa. The estimated age of their common ancestor, the root of haplogroup N, is around 55–65 kya [13].

The mitochondrial haplogroup N1a is widely accepted as a molecular signal of Neolithic migrations of the first farmers from the Near East into Europe around 7500 years ago. In modern day populations, its frequency is very low, usually around 0.2%, but it has been detected in up to 25% of ancient human remains from the Neolithic period in Central Europe [14]. In spite of its rare occurrence in modern-day populations, the N1a haplogroup shows a wide distribution and a deep diversity within its sublineages [13,15]. Based on its distinct mutations, three phylogeographic branches were proposed, the European, Central Asian, and African/South Asian lineage, presumed to be absent in contemporary European populations [14]. Haplogroup X, also a rare mitochondrial haplogroup, originated in the Near East ca. 30 kya, is divided into four distinct subhaplogroups, denoted as X1–X4. The predominant lineage is X2, while others are rarely found, usually in Near Eastern and North African populations [16,17]. Haplogroup HV, as the major subclade of R0, is also rare or absent in north and west European populations, but it is more common in southern European regions. Its frequency reaches 7% to 9% in certain Italian populations, but it is more prevalent in the Near East and the Caucasus [18]. A recent study of the rare human mitochondrial haplogroup HV reported a surprisingly large number of novel lineages, but HV2b remained unrecognized [19].

In order to shed additional light on those rare and less studied parts of mitochondrial phylogenetic trees, we integrated the results of the NGS sequencing data with our previously sequenced similar mitogenomes and joined them with all the publicly available data in the attempt to refine the global phylogeny of the N1a, X, and HV2 haplogroups.

## 2. Materials and Methods

### 2.1. Sample Collection and DNA Extraction

Samples were collected within several projects approved by local Ethical Committees, and all individuals gave their informed consent according to the international standards in research involving human DNA. DNA extraction from whole blood was performed in the Laboratory for Molecular Anthropology of the Institute for Anthropological Research in Zagreb and in the Institute of Genomics, Tartu, Estonia, using both the salting out procedure [20] and the NucleoSpin Blood kit (Macherey-Nagel, Dueren, Germany) according to the manufacturer’s instructions.

**Figure 1:**
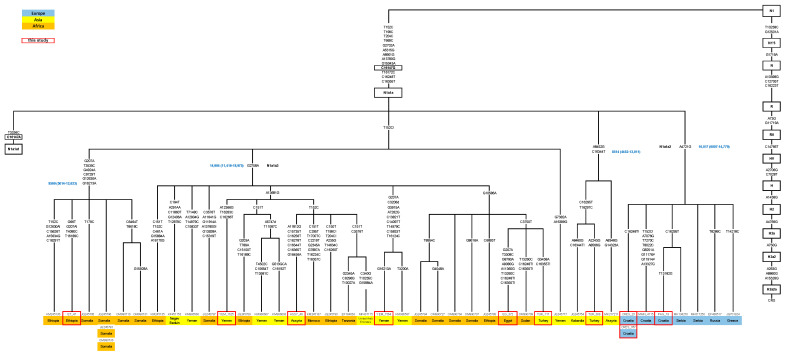
Maximum parsimony phylogenetic tree of the complete mtDNA sequences belonging to the haplogroup N1a1a. The numbers on the branches refer to the substitutions relative to rCRS [21]. The samples from the present study are labeled as shown in Supplementary Table S3, and for the published data, the GenBank accession number is indicated. Coalescence age estimates, expressed in years and highlighted in blue, are shown on the branches and were calculated using the mutation rate based on the mtDNA complete genome variability data [22].

**Figure 2:**
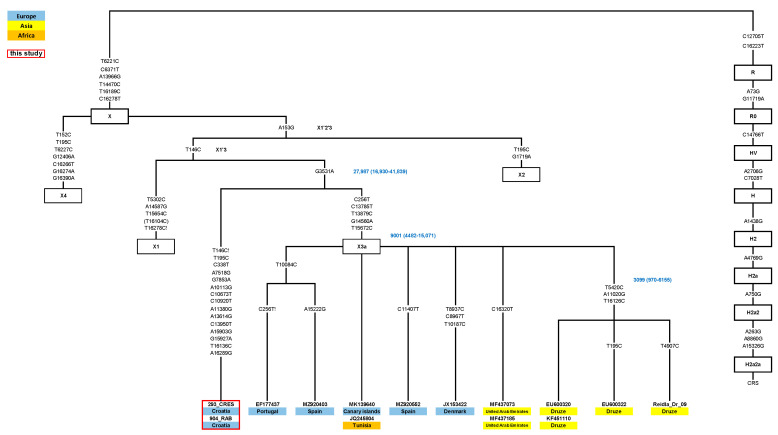
Newly constructed phylogenetic tree of the X1’3 branch. The designations are the same as in Figure 1.

**Figure 3:**
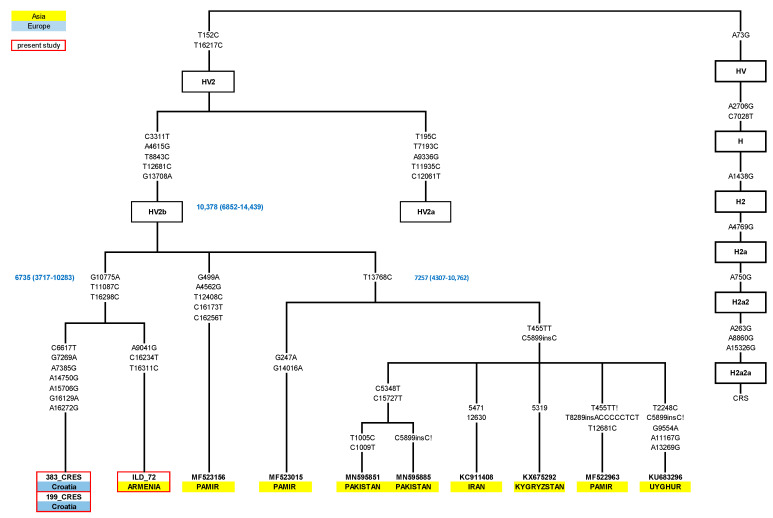
Newly constructed phylogenetic tree of the HV2b branch. The designations are the same as in Figure 1.

**Figure 4:**
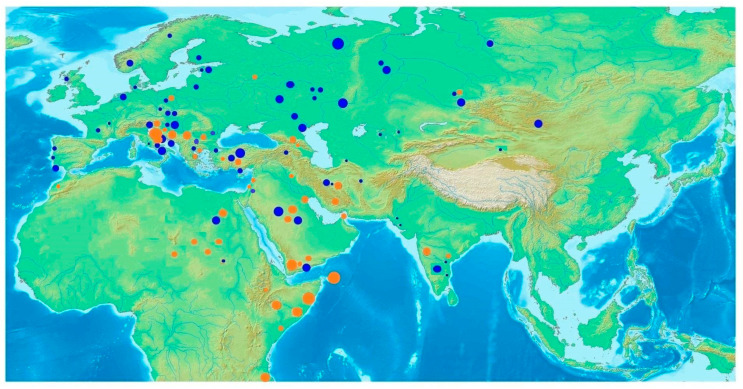
World distribution of the N1a1a (16147G) variant, marked in orange, and the N1a1a1 (16147A) variant, marked in blue.

In total, 32 samples affiliated to rare haplogroups for the Croatian population, according to the HVS region, were chosen from the mtDNA database of the Institute of Anthropological Research to be analyzed using next-generation sequencing. Out of these 32 samples, 7 samples indicating a novel finding were chosen and integrated with 9 of our previously completely sequenced samples and samples from the literature to reconstruct the current phylogenetic trees of haplogroups N1a, X, and HV2. Therefore, the trees are based on 68 complete mitogenomes—16 new, yet unreported, sequences from the Croatian and Estonian database and 52 samples reported in the literature (Figure 1, Figure 2 and Figure 3. Additionally, a world distribution map was created for two N1a variants (Figure 4).

### 2.2. Haplogroup Affiliation and Sequencing

Haplogroup and subhaplogroup affiliations were previously determined based on single-nucleotide polymorphisms from the coding and noncoding regions of the mitochondrial genome, according to the PhyloTree build 17. The HVS-I sequences were aligned and analyzed according to the Revised Cambridge Reference Sequence (rCRS, NC_012920) [21,23] by using ChromasPro software 2.6.6. (Technelysium Pty Ltd., Tewantin, QL, Australia).

The initial complete sequencing of 9 mitochondrial genomes was performed at the Estonian Biocentre, Institute of Genomics, Tartu, Estonia using Sanger sequencing. Sequencing was performed on the Applied Biosystems 3730xl DNA Analyzer, Thermo Fischer Scientific, Waltham, MA, USA) with the BigDye Terminator sequencing kit (Applied Biosystems, Warrington, UK), according to the previously published methodology [24,25].

Target enrichment, library preparation, and sequencing of the selected subsample (*n* = 32) were performed at the Forensic Science Centre “Ivan Vučetić”, Zagreb, Croatia according to Illumina 2016 Protocol, with the modifications described previously [26,27]. Briefly, the samples were amplified by a long-range PCR with PrimeSTAR^®^ GXL DNA polymerase in two amplicons for each sample. The conditions of the optimized protocol for both fragments were as follows: 9.1 kb fragment: 25× (98 °C 10 s, 60 °C 15 s, 68 °C 9 min, 6 s); and 11.2 kb fragment: 25× (98 °C 10 s, 68 °C 10 min). Both mtDNA amplicons were then quantified on a QubitTM 3.0 Fluorometer (Thermo Fisher Scientific) with the QubitTM dsDNA High Sensitivity kit. Both mtDNA amplicons were normalized to equal concentrations of 0.2 ng/μL, as described in the Illumina^®^ protocol, and pooled for each sample, wherefrom the volume of 5 μL was taken for library preparation (i.e., total input of 1 ng). Nextera^®^ XT Library Prep Kit was used according to the Illumina^®^ protocol, and negative controls (reagent blanks) were used in the target enrichment (NC-PCR) and library preparation (NC-LIB) procedures [28]. The purification of libraries was performed with Agencourt AMPure XP magnetic beads (Beckman Coulter, Brea, CA, USA). The libraries underwent quantification with LabChip^®^ DNA High Sensitivity Assay on LabChip^®^ GX Touch HT (PerkinElmer, Waltham, MA, USA) and were diluted to concentrations of 0.2 ng/μL. The libraries normalized to 4 nM were pooled, denatured, and diluted to 12 pM, with 5% PhiX Sequencing Control v. 3 (Illumina, San Diego, CA, USA) spike-in (Illumina^®^ Denature and Dilute Libraries Guide for MiSeq System). The paired-end sequencing of all 32 samples was performed within a single run on an Illumina^®^ MiSeq FGx^TM^ instrument by using the MiSeq^®^ Reagent Kit v.2, 300 cycles (2 × 151 bp). The run quality metrics were reviewed in Illumina^®^ Sequencing Analysis Viewer (SAV) v.1.11.1 software: a cluster density of 1494 K/mm^2^, with 87.2% of clusters passing the filter was reported, and 78.8% of the sequenced bases exhibited a base call quality score (Q) above 30.

The raw fastq reads generated by the MiSeq^®^ Reporter v.2.5.1.3 (Illumina^®^) were mapped to the rCRS (NC_012920) [21,22] using the bwa program version 0.7.12-r1039 [29], with the ‘mem’ algorithm and default parameters. Samtools v1.8 [30] was used to convert the mapped reads to BAM format, fixing mate-pair information, and sorting and indexing the resulting files. The indels were left-aligned with the GATK v4.0.3.0 [31]. LeftAlignIndels command against the rCRS reference fasta file, and subsequently the PCR duplicates were removed with Samtools. The reads were filtered using Bcftools v1.8 [32], with a minimum base quality of 20 and a mapping quality of 30. The base frequencies at each reference position were counted into a VCF file, normalized by left-aligning indels, and the multiallelic variants were split into separate rows, producing the final data format for manual review. The coverage across all samples and positions after PCR duplicate removal amounted to 4243 ± 2253 (mean ± standard deviation). All reported variants were manually reviewed by inspecting the respective BAM files in the Integrative Genomics Viewer (IGV) tool v.2.4.16 [33,34]. The haplogroup and subhaplogroup affiliations were determined based on the full haplotypes, by using HaploGrep2 v.2.1.1 [35]. Computationally assigned haplogroups were manually validated against the PhyloTree, Build 17 [36].

### 2.3. Coalescence Time Estimates and Phylogenetic Trees Reconstruction

Coalescence time estimates were computed with the Bayesian MCMC approach implemented in the BEAST v1.7.5 suite of software [37]. All analyses were performed using the HKY model of nucleotide substitution [38]. Rate variation among sites was modeled using a gamma distribution with four rate categories. A strict clock model was used as the clock model [39]. The tree prior used was a piecewise-linear Bayesian skyline model [40]. The prior normal distribution for the mutation rate was set based on [22] Soares et al., 2009. Each BEAST run was performed in the same way: a single MCMC chain was run for 50,000,000 steps, sampled every 1000 steps, and the first 10% was discarded as a burn-in. Appropriate effective sample size values (ESS > 200) for each parameter in the model were checked in Tracer v1.5. As the BEAST v1.7.5 software assumes a linear mutation rate, the time estimates obtained from the BEAST v1.7.5 analyses were corrected by the published formula [22] (Supplementary Table S2).

The maximum parsimony phylogenetic tree was first constructed with the software mtPhyl 5.003 (URL: https://sites.google.com/site/mtphyl/home, accessed on 23 February 2023), and the tree topologies were subsequently manually verified, by using all publicly available sequences from the current literature and Mitomap up to March 2023. The trees of the complete mtDNA sequences (52 published and 16 novel ones—9 from our database + 7 sequences from the NGS dataset) were rooted to the Revised Cambridge Reference Sequence (rCRS), in order to enable comparison to the previously published data.

A world distribution map was created for both the N1a 16147A and 16147G variants (Figure 4, with all the available data from the published literature (Supplementary Table S1). We included only those populations harboring at least one individual with an N1a haplogroup. Each N1a variant is represented in a different color—16147A variant in blue and 16147G in orange, while the frequency of the N1a samples in each population is directly proportional to the node size. The N1a 16147A distribution is based on 70 populations from the literature and the present study, with 165 out of 25,537 different samples. The N1a 16147G variant distribution map is based on 43 different populations, with 96 out of 14,822 different samples (Supplementary Table S1).

## 3. Results

In order to expand the current Croatian forensic database and achieve a fully representative database, 32 full mitogenomes of rare mtDNA lineages from Croatian island and mainland populations were chosen from our larger mtDNA database and sequenced on the Illumina platform. Their haplogroup affiliations, frequencies, and mutations are presented in Supplementary Figure S1 and Supplementary Table S4. All those sequenced samples belong to the mtDNA clades D, F, HV, I, L, N, W, X, U1, U3, U7, and U8, all present in less than 5% of the Croatian population. Based on their unique mutations, as seen by comparison with the Croatian and Estonian mtDNA databases, we selected 7 out of 32 samples for further analysis of the N1a, X, and HV haplogroups. Those seven samples of Croatian origin, together with nine previously sequenced samples of different origin from our database (N = 16 in total), were selected for the reconstruction of phylogenetic trees of haplogroups N1a, X, and HV2. In the reconstruction of the phylogenetic treesthirty-two published N1a1a sequences, twelve X3a, and eight HV2b from the literature were also used (52 in total). All the new and published sequences (with sample ID, population of origin, reference, and mutations) that were used in the reconstruction of the trees are presented in Supplementary Table S3 and Figure 1, Figure 2 and Figure 3.

### 3.1. Reconstruction of the Maximum Parsimony Phylogenetic Tree of the N1a1a Ancestral Variant

In order to shed new light on the ancestral branch of the N1a 16147G variant and to clarify its spread across the Old World, we reconstructed the tree of the N1a 16147G variant using 32 published [10,11,13,19,41,42,43,44,45,46,47] and 11 new complete sequences (Figure 1, Supplementary Table S3). In order to reconstruct an unbiased phylogeny of the so-called “African/South Asian branch”, we chose sequencing samples from various regions in Africa, Middle and Near East, as well as from Europe. Three distinct clusters were recognized. The largest part of the newly reconstructed tree of the N1a 16147G variant was marked by a HVSII back mutation at the position 152. A specific African branch was observed within this large cluster, marked by transitions in the positions 207, 3535, 4924, 9729, 12630, and 16213. Its estimated age was 8569 years (5014–12,623). The other part of this cluster, encompassing the vast majority of the published samples and showing a deep diversity mostly within North and East Africa, the Arabian Peninsula, and the Near East, was characterized by the mutation 2758 (N1a1a3), and it is also the oldest part of this branch, with an estimated age of 14,904 years (11,416–18,975) [13,48]. Several distinct groups were visible within this cluster. The first two were characterized by the mutations 13681 and 10586, and the smallest one was marked by nine mutations shared by two Yemeni samples. It is expected that this cluster will expand with additional sequences in the future. 

Besides the largest cluster marked by the 152 back mutation, two smaller clusters stemming directly from the N1a1a branch arose as well. Three Near Eastern samples were joined in a separate cluster marked by transitions at positions 8452 and 16344, with an age estimate of 8514 years (4452–13,011). The second cluster marked by a 4721 transition encompassed eight different complete sequences from various European regions—four samples of Russian, Greek, and Serbian origin [10,11,49], joined with four new sequences from different parts of the Croatian territory. One sample originated from the continental mountainous part of Croatia, while three others were from the Adriatic islands of Cres and Pag. The coalescent time estimation of this European cluster was estimated at around 10,017 years (6007–14,779).

In the attempt to trace the geographical distribution of the contemporary N1a variants on a global scale and to revise the generally accepted postulates of their spatial predominance, we created world distribution map for both the N1a 16147A and 16147G variant (depicted in Figure 4) with all the available data from the literature on populations of European, Asian, and African origin (Supplementary Table S1). Only those populations harboring at least one individual with 16147A or 16147G variants were presented in blue and orange colors, respectively. The frequency of the N1a samples in each population was directly proportional to the node size. In spite of the current belief that both variants are quite rare and continentally specific, their dispersals and variety revealed much wider distribution and frequency than was previously known.

### 3.2. Reconstruction of the Maximum Parsimony Phylogenetic Tree of the X1’3 Haplogroup

We also reconstructed the phylogenetic tree of the X1’3 using twelve published [13,16,17,43,46,50,51,52,53] and two new complete sequences (Figure 3, Supplementary Table S3). The complete sequencing of samples assigned to the X* haplogroup with the 16136-16189-16223-16278-16289 HVSI motif did not allow their affiliation to any known subhaplogroup on the X phylogenetic tree and suggested an individual twig. They were joined more closely with the X3 subclade by a common transition 3531, but they otherwise represented a completely new lineage. The cluster marked by the 3531 mutation showed a very old age—27,987 years (16,930–41,839), while the X3a cluster was much younger, estimated at 9001 years (4482–15,071).

### 3.3. Reconstruction of the Maximum Parsimony Phylogenetic Tree of the Newly Proposed HV2b Haplogroup

The sequencing of two HV2 samples from the Cres Island was also performed and joined with a sample of Armenian origin from the Estonian database, sharing a similar HVSI motif. In the attempt to clarify their unusual connection, we constructed the maximum parsimony phylogenetic tree using eight complete mtDNA sequences from the literature and Mitomap [54,55,56,57,58] and three new samples (Figure 3, Supplementary Table S3). We can conclude based on our findings that we identified a novel HV2 subbranch, which we named HV2b. The HV2b branch was marked by transitions in the positions 3311, 4615, 8843, 12681, and 13708, estimated at around 10,378 years (6852–14,439), encompassing at least two different clusters. The largest group, marked by the mutation 13768, was around 7257 years old (4307–10,762), while the newly sequenced samples of the Croatian and Armenian cluster formed a separate group with an age estimate of 6735 years (3717–10,283).

## 4. Discussion

The Croatian Adriatic islands serve as genetic isolates characterized by the presence of atypical lineages when compared to the contemporary European population, which are more prevalent and frequent due to the influence of genetic drift and recurrent bottlenecks. Incorporating these rare mitogenomes into the EMPOP database would enhance its forensic power by facilitating the identification and matching of individuals possessing uncommon genetic profiles for a particular region. Furthermore, the identification of novel branches within certain haplogroups, such as the HV2, X1’3, and N1a1a ancestral variant, underlines the importance of exploring isolated populations and their distinctive characteristics in shaping the contemporary structure of the human population. Therefore, the objective of this study was to reconstruct the phylogenetic trees for these haplogroups by sequencing rare mitochondrial haplotypes obtained from our database. This research provides valuable insights into the genetic diversity and evolutionary history of these specific lineages, contributing to our understanding of the broader human population structure.

### 4.1. New Insight into the Phylogeny and Phylogeography of the Ancestral N1a 16147G Variant—European Cluster inside the African/South Asian Branch

The west-Eurasian mitochondrial haplogroup diversity encompasses haplogroups mainly derived from haplogroup R, the daughter branch of the macrohaplogroup N. The estimated origin of haplogroup N was most likely in the Arabian Peninsula around 55–65 kya, shortly after the Out-of-Africa migration, where the derivation into the R clade took place around 59 kya [13]. West Eurasian mitochondrial haplogroups N1 (including I), N2 (including W), and X stem directly from the N node [59]. Due to their common features—relative rarity (usually below 5.0% in European populations) and scattered distribution, the members of these clades have generally been neglected in past studies. The N1a haplogroup shows a wide distribution and a deep diversity within its sublineages [13,15,47]. Based on the N1a haplogroup differences, three distinct phylogeographic branches—European (16147A variant), Central Asian (16147A variant), and African/South Asian (16147G variant)—were proposed by Haak et al., 2005 [14]. The vast majority of ancient and modern N1a lineages across Europe and Central Asia form a European/Central Asian branch characterized by the 16147A mutation. Although its impact on the modern genetic pool and its dispersal routes are still highly debated, findings of the diverse mitochondrial N1a lineage marked by the 16147A mutation (up to 25%) among ancient human remains associated with the Linear pottery culture and Alföld Linear Pottery culture were connected with the spread of the first farmers into Central Europe 7500 years ago [14]. Contemporary western Eurasian populations usually harbor below 0.2% of N1a 16147A variant, and this drastic decline of this haplogroup in modern-day populations has not been fully clarified. Based on a large dataset of early Neolithic skeletons, the presence of the N1a 16147A variant in early farmers from the Carpathian Basin (6.82–10.26%) and Central Europe (12.04%) affirmed its role as a marker for the Continental route of the Neolithic expansion [60,61,62]. This variant of N1a was also found in one of three individuals of the Megalith culture in Southwestern France, indicating its spread to the remote parts of the European continent [63], but it has not been recorded in the Mediterranean region [64,65,66]. The world distribution of the N1a 16147A variant in this study showed that the distribution of the so-called European/Central Asian branch spreads from the westernmost part of Europe all the way to Eastern and Southern Asia and from Northern Eurasia all the way to Northern Africa. Surprisingly high frequencies of this N1a 16147A variant were found around the Near East and Arabian Peninsula. The highest frequency of the N1a 16147A variant on the European territory was located around the central European region, which served as a migratory route for Neolithic farmers. Other parts of Europe did not show a substantial prevalence of this lineage.

The African/South Asian branch, characterized by the 16147G mutation, was believed to be more common in the Arabian Peninsula, northern Africa, and the Near Eastern region and very rare, or even absent in Europe [15,67,68]. It has also not been found in any Neolithic excavation site in Europe. This suggests that the initial diversification of the ancestral N1a branch took place in the Southern Arabian Peninsula shortly after the Out-of-Africa migration, with substantial back-to-Africa migratory routes mostly through the Eastern African coast and Northern African regions, to a lesser extent [13]. The highest reported finding (6.2%) of the 16147G N1a variant in this region was reported in the Soqotra archipelago, an isolated group of islands situated between the Horn of Africa and southern Arabia on the proposed route of the ancient gene flow across the Red Sea [69]. As depicted in the world distribution map, it showed a substantially different distribution than reported so far. Apart from the previously reported African and Middle Eastern regions, members of this rare clade were found to be scattered globally, from central and Southeastern Europe to Eastern Asia and India. It also showed a significant presence in Europe as well, especially in Croatia and other South Eastern European countries, such as Bosnia and Herzegovina, Bulgaria, Serbia, and Greece [70,71,72]. Moreover, a substantial frequency of the N1a 16147G variant was detected in the Adriatic/Southeastern European region (Figure 2). It is noteworthy that the highest observed frequency of both N1a clades was reported in small and isolated populations, such as the Adriatic islands [5], the Soqotra islands [69], and the Komi population [73], with over 9, 6, and 17%, respectively. To the best of our knowledge and probably due to genetic drift and small population size, this is the highest N1a frequency in any modern human population. Our calculation of the diversification time of lineages within the novel European cluster (10,017 years ago) corresponds with ancestral variants of this haplogroup, suggesting an even older presence in Europe than Neolithic variants (Supplementary Table S2).

### 4.2. Complete Sequencing of the Rare X* Lineage Revealed a Novel Branch within the X Haplogroup Phylogeny

Haplogroup X was estimated to originate in the Near East ca. 30 kya. According to the current nomenclature it is divided into four subclades denoted as X1–X4. The X2 subclade encompasses by far the largest part of the X tree, marked by both the highest frequency and diversity of all X lineages scattered worldwide It is geographically distributed among West Eurasians, northern groups of Native Americans, as well as in northern Africa and the Near East, but with low frequency, around 1–2% [13,16]. Some of X2 branches are predominant in the Near Eastern and North African region, while the others are restricted to certain Native American or European populations [8,74]. Although high global genetic diversity has been reported for haplogroup X2, this haplogroup is far less common in Croatian insular populations according to our previous findings. Subclades X1 and X4 are mainly found in Near East and North Africa, while X3 has mostly Near Eastern and Mediterranean distribution [13,17]. Our samples from two Croatian Adriatic Islands (Cres and Rab), representing identical lineages, revealed a novel individual twig in the X haplogroup phylogeny. This branch is connected with the X3 haplogroup by a common mutation 3531 in the coding region of the mitochondrial genome. This mutation was previously recognized as defining for the X3 branch, and our novel lineage shares this, but not the other defining mutations. Capturing such rare mitochondrial lineages in the sieve of sampling methods is clearly an advantage of studying isolated island populations as reservoirs of ancient diversity in the study of contemporary human populations.

### 4.3. An Unusual Genetic Link between Adriatic and Near Eastern Populations within the HV2 Haplogroup

Similar interesting trace of a long-distance migration from Near/Middle East in the Adriatic gene pool was found in the HV2 haplogroup. Haplogroup HV is predominantly present in the Near East, Middle East, and in the Caucasus, while in Europe it is spread unevenly—it is rare or absent in the north and west, but more common among southern and eastern Europeans [19]. A revised topology of haplogroup HV based on 316 novel and previously published complete mitochondrial genomes [18] defined the HV2 haplogroup only by the unstable 73 mutation and the HV2a haplogroup with several other mutations, including 16217 position in the HVSI region of the mitochondrial genome. Haplogroup HV2 has been dated at 36–42 kya and most likely arose in Iran between the time of the first settlement by modern humans and the LGM [54]. Our complete sequencing revealed a novel HV2 subbranch, which we dubbed HV2b (Figure 3). Most members of this novel clade belong to Central Asia (Pamir (China), Kyrgyzstan) and the Middle East (Iran, Armenia). Finding of a specific cluster within the novel HV2b clade, encompassing a sample from the Adriatic and from Armenia, suggests possible one time long-distance migration in the past. 

## 5. Conclusions

The identification of a previously unknown European cluster within the African/South Asian N1a 16147G branch suggests the emergence of a new founder lineage likely originating locally within European territories, potentially predating the Neolithic period. Additionally, the wider distribution of N1a 16147A than previously reported in the literature suggests a need for a revision of the complete N1a phylogeny. Furthermore, the detection of rare mitochondrial lineages, such as X* and HV2b, indicates the presence of signals with origins in the Near/Middle East within the Adriatic gene pool. These findings highlight that small human genetic isolates can serve as reservoirs of population variability, offering insights into prehistoric migratory events that have played a significant role in shaping the maternal genetic landscape on a larger scale.

## Data Availability

The data that support the findings of this study are available from the corresponding author, upon request.

## Supplementary Materials

The following supporting information can be downloaded at: https://www.mdpi.com/article/10.3390/genes14081614/s1, Figure S1: Rare Croatian haplogroups with observed frequencies; Table S1: A list of all published N1aA and N1aG HVS regions with frequencies [75,76,77,78,79,80,81,82,83,84,85,86,87,88,89,90,91,92,93,94,95,96,97,98,99,100,101,102,103,104,105,106,107,108,109,110,111,112,113,114,115,116,117,118,119,120,121,122,123,124,125,126]; Table S2: Coalescence time estimates for the subhaplogroups of N1a, X and HV2; Table S3: New phylogenetic trees of N1a, X, and HV2 with fasta files; Table S4: List of all samples sequenced using next generation sequencing, with detected mutations.
