# Supplementary figures and images for: Refining the Global Phylogeny of Mitochondrial N1a, X, and HV2 Haplogroups Based on Rare Mitogenomes from Croatian Isolates
Source: Genes (Basel). 2023 Aug 12;14(8):1614. doi: 10.3390/genes14081614 (PMC10454736; doi:10.3390/genes14081614)

**Supplementary Figure S1. The observed frequency distribution of 32 rare Croatian haplogroups.**

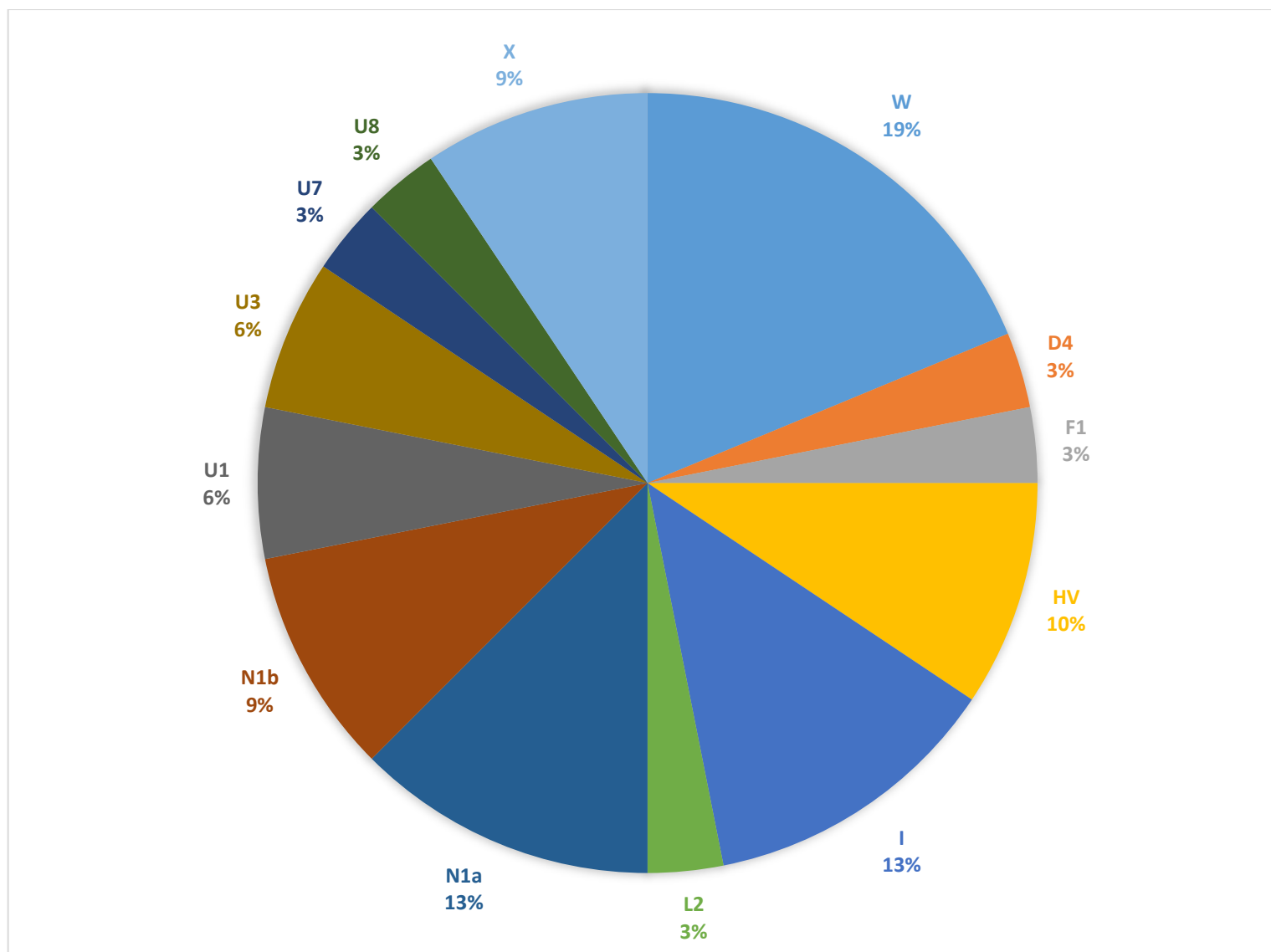

Supplement: Supplementary file 1 [file genes-14-01614-s001.zip › Supplementary Figure S1.pdf]
